# Diagnostic Power of the Fibrinogen-to-Albumin Ratio for Estimating Malignancy in Patients with Adnexal Masses: A Methodological Study

**DOI:** 10.3390/diagnostics15182372

**Published:** 2025-09-18

**Authors:** Gözde Şahin, Ayşe HazırBulan, Hatice Argun Atalmış, İlkbal Temel Yüksel, Işık Sözen, Alper Koçbıyık, Nilüfer Çetinkaya Kocadal, İsmet Alkış

**Affiliations:** 1 Gynecologic Oncology Clinic, Basaksehir Cam and Sakura City Hospital, Istanbul 34480, Turkey; aysehazirbulan@gmail.com (A.H.); haticeargunatalmis2@gmail.com (H.A.A.); drilkbaltemel@gmail.com (İ.T.Y.); isiksozen@gmail.com (I.S.); cetinkayanilufer@gmail.com (N.Ç.K.); ismetalkis@hotmail.com (İ.A.); 2 Patology Clinic, Basaksehir Cam and Sakura City Hospital, Istanbul 34480, Turkey; alperkocbiyik@gmail.com

**Keywords:** adnexal masses, borderline, benign, malignant, fibrinogen-to-albumin ratio, biomarker

## Abstract

**Background**: Adnexal masses are common in women across different age and hormonal states: pregnancy, premenopause, and postmenopause. Ovarian carcinoma, a malignancy arising in the adnexa, poses significant health risks. While malignancy risk increases with age and postmenopausal status, current methods for stratifying borderline cases remain inadequate, potentially leading to over- or undertreatment that may affect fertility or survival. **Methods**: This retrospective study was conducted with 318 adult women who were diagnosed with adnexal masses and underwent surgery at a university hospital between 2020 and November 2023. Patient data were retrieved from the hospital’s electronic medical record system. Routinely measured preoperative serologic parameters—carbohydrate antigen (CA)125, CA19-9, CA15-3, carcinoembryonic antigen (CEA), Alpha-fetoprotein (AFP), Lactate dehydrogenase (LDH), and fibrinogen-to-albumin ratio (FAR) levels—were analyzed alongside final histopathological results. No procedures outside routine clinical practice were performed. Diagnostic performance of each marker was evaluated using receiver operating curve (ROC) analysis. **Results:** A total of 318 patients with adnexal masses were analyzed. The FAR levels were significantly elevated in malignant compared to borderline and benign groups (*p* < 0.001), and FAR alone showed 47% sensitivity and 91% specificity for borderline tumors, whereas CA125 showed 70% sensitivity and 85% specificity. Multivariate models combining FAR, CA125, and CA15-3 achieved the highest diagnostic accuracy, with superior AUCs compared to single biomarkers. **Conclusions**: FAR is a simple, accessible inflammatory marker that complements CA125 by enhancing specificity. Combination of multiple markers with the highest sensitivity and specificity, together with FAR, may reduce the risk of both false negatives, offering a more balanced and accurate diagnostic tool for preoperative stratification of borderline tumor cases.

## 1. Introduction

Adnexal masses are common in women, with reported prevalence rates of 0.05–2.4% during pregnancy [1], 152 per 100,000 in the postmenopausal period [2,3], and 2.6 per 100,000 in adolescence [2,4,5]. The majority of the lesions, whether in premenarcheal, reproductive-age, or postmenopausal women, are benign [4,6]. While the risk for malignancy increases with age, 7.7% of cases were classified as borderline malignant in a large cohort study [2]. In reproductive-age women, 5–10% of ovarian masses ultimately prove malignant [6], and even in pediatric and adolescent populations, most adnexal neoplasms remain benign [4,5,7]. However, 15–20% of epithelial ovarian malignancies were defined as ‘hard to stratify’ borderline cases [8], and it is crucial to improve the classification of such cases to prevent under- or overtreatment [8,9]. Nevertheless, any persistent or enlarging mass under surveillance must be treated as potentially malignant until proven otherwise.

Clinical presentation of adnexal mass is often nonspecific, with abdominal distension, pain or discomfort, pelvic pressure, urinary or gastrointestinal symptoms, and acute events [3,10,11]. Ultrasound and histopathologic tools are the hallmarks of stratification using risk assessment systems [1,12,13,14,15]. These systems evaluate the masses based on morphology, vascularity, and bilaterality. A combined approach with imaging and histopathology yields 80-95% accuracy in detecting malignancy [14,15]; however, borderline cases require additional examination, such as of levels of serologic markers against tumors [16]. Conventional biomarkers, including carbohydrate antigen (CA)125, CA19-9, CA15-3, carcinoembryonic antigen (CEA), Alpha-fetoprotein (AFP), Lactate dehydrogenase (LDH), Inhibin A/B, Beta-Human Chorionic Gonadotropin (β-hCG), and Human Epididymis Protein 4 (HE4), are often used to estimate malignancy [17]. However, the preoperative diagnosis of borderline cases remained challenging due to the lack of association between serologic marker concentrations, lesion size, and histopathology [18]. Even the assessment of borderline cases by frozen sections collected from intraoperative tissue, using CA125, CA15-3, CA19-9, and CEA, remained inconclusive [19,20].

Fibrinogen is a liver-derived glycoprotein released in response to systemic inflammation as an acute-phase protein (APP) from the liver [21,22]. Fibrinogen is important for coagulation, thrombosis, and regulation of inflammation [23], closely working with the immunological network of pro-inflammatory cytokines—Interleukin-6 (IL-6), Interleukin-1 beta (IL-1β), and Tumor Necrosis Factor-alpha (TNF-α) [24,25]. Fibrinogen not only reflects systemic inflammation but may also be produced by tumor cells themselves to promote angiogenesis in the tumor microenvironment. Hence, elevated fibrinogen levels correlate with worse outcomes in solid tumors [23]. Serum albumin is also a liver-produced protein. Conversely, a reduced albumin level is associated with inflammation and other disease pathologies recognized as negative APP [26]. The fibrinogen-to-albumin ratio (FAR) has emerged as a prognostic marker in cardiac disorders [27,28], different types of malignant tumors [29], pancreatic cancer [30], gallbladder cancer [31], and type 2 diabetes-induced kidney disease [32], yet its diagnostic and prognostic value in adnexal masses has not been systematically explored.

Furthermore, a large external validation study of ultrasound-based risk stratification systems (O-RADS, GI-RADS, and ADNEX) was conducted on 734 patients with adnexal masses (including 69 borderline cases), and reported the diagnostic challenge posed by borderline lesions even with advanced imaging systems [33]. While imaging-based models such as O-RADS, ADNEX, and GI-RADS are well-established in clinical practice, integrating FAR with these models could enhance diagnostic accuracy. For instance, studies have shown that combining O-RADS with CA125 improves diagnostic performance, suggesting that integrating biomarkers like FAR could further refine risk stratification [34]. Similarly, the ADNEX model, which classifies masses based on multiple parameters, has demonstrated high diagnostic accuracy [35]. Incorporating FAR into such multifactorial models may provide additional prognostic information, particularly in distinguishing between benign and malignant lesions. Furthermore, while GI-RADS offers a structured approach to evaluating adnexal masses, its integration with serum biomarkers like FAR could potentially improve its diagnostic precision [36].

A recent meta-analysis study reported that a high FAR is significantly associated with poorer overall survival (OS) and progression-free survival (PFS) outcomes in gynecological malignancies including ovarian cancer [37]. Moreover, in advanced epithelial ovarian cancer cohorts, a low preoperative albumin-to-fibrinogen ratio (AFR, inverse to FAR) was independently associated with poorer PFS and OS, with ROC-derived cutoffs demonstrating strong prognostic discrimination (AUC = 0.773) [38]. One diagnostic study assessed the value of FAR in combination with CA125, HE4, the systemic immune–inflammation index (SII), and the prognostic nutritional index (PNI), showing that the multimarker model including FAR provided better sensitivity (84%) and overall accuracy (86%) than CA125 alone for distinguishing ovarian malignancy preoperatively [39]. A diagnostic nomogram study, assessing age, CA125, FAR, the monocyte-to-lymphocyte ratio, and ultrasound findings, achieved excellent preoperative discrimination between malignant and benign ovarian masses (AUC = 0.94), outperforming established models and showing robust calibration and decision-curve performance [40]. These findings suggest that FAR may add unique diagnostic and prognostic value beyond conventional markers and risk models by integrating inflammatory and nutritional status indicators to enhance stratification, especially in borderline adnexal lesions.

Our aim is to determine the diagnostic and prognostic significance of FAR and other tumor markers in patients with adnexal masses. Thus, we aimed to determine whether this inflammation-based index can improve preoperative stratification, particularly for borderline cases, thereby guiding surgical planning, informing surveillance strategies, and ultimately impacting patient morbidity and mortality.

## 2. Materials and Methods

### 2.1. Study Design and Setting

This diagnostic methodological study was a retrospective study conducted with patients who had adnexal mass diagnoses in the Gynecological Oncology Clinic at a university hospital between May 2020 and November 2023. The study was carried out according to the Declaration of Helsinki and the Strengthening the Reporting of Observational Studies in Epidemiology (STROBE) guidelines.

### 2.2. Study Population

In the study population, we included adult women patients who were (1) diagnosed with an adnexal mass and (2) had previously been included. Patients who (1) had comorbidities such as endometrium cancer, cervix cancer, and vulva and vagina cancer in addition to adnexal mass, (2) were diagnosed with other types of carcinomas, (3) were presenting with diffuse peritoneal ascites, (4) underwent hysterectomy due to benign tumors, (5) had relapsed ovarian tumors, (6) were presenting with recurring cysts, or (7) had missing data or unavailable cytological or histopathological results were excluded from the study.

All eligible cases were consecutively included in the study. No formal power calculation was performed. Patients with missing data were excluded from the analysis.

### 2.3. Data and Variables

The adnexal mass diagnoses, follow-up, and treatment were determined according to the NCCN guideline [41] and ESGO/ISUOG/IOTA/ESGE consensus [10]. The mass size, characteristics, and disease staging were defined according to the FIGO scale system [42]. The borderline, benign, and malignant stratification (the two reference categories) was determined according to the ESGO/ISUOG/IOTA/ESGE consensus. The tumor size was measured from the postoperative specimen. The histopathological tumor stratification was performed according to the WHO guidelines [43]. Peritoneal ascites samples of patients were used for the preoperative cytology analysis. Laboratory analysis of markers and chemicals was performed on the standardized preoperative blood samples collected within 24 h prior to surgery. Serologic parameters, CA125, CA19-9, CA15-3, CEA, AFP, LDH, and procalcitonin levels, were compared among benign, borderline, and malignant case groups, with the benign group representing the baseline level. FAR is calculated as the preoperative serum fibrinogen (g/L) divided by albumin (g/L) multiplied by 100. The accuracy and sensitivity of conventional tumor markers CA125, CA19-9, CA15-3, and CEA vs. FAR in stratifying borderline cases were determined based on ROC analysis. The cut-off values determined specifically for the study are as follows: CA125 > 49.9 U/mL; CA19-9 > 34.8 U/mL; CA15-3 > 29.8 U/mL; CEA > 1.4 ng/mL; AFP > 7 ng/mL; LDH > 211 U/L; and procalcitonin > 0.5 ng/mL. Meanwhile, a FAR cut-off > 11.51, which was determined based on ROC curve analysis in our cohort, distinguished borderline/malignant from benign.

### 2.4. Statistical Analysis

Since we intended to enroll all eligible patients in the study according to the inclusion and exclusion criteria, a minimum sample size was not calculated. Statistical analyses were performed using IBM SPSS Statistics for Windows, version 27.0 (IBM Corp., Armonk, NY, USA), and MedCalc version 16 (MedCalc Software bvba, Ostend, Belgium). Kolmogorov–Smirnov and Shapiro–Wilk tests were used to assess the normality of the data. Descriptive statistics are presented as the median with the interquartile range (IQR) for non-normally distributed numerical data and the frequency (n) and the percentage (%) for categorical data. The diagnostic performance of tumor markers in estimating borderline/malignant tumors, and only malignant tumors, was analyzed using receiver operating characteristic (ROC) analysis. To evaluate the combined predictive performance of the selected biomarkers, we constructed logistic regression models. The three biomarkers which have the highest statistically significant diagnostic performance individually (FAR, CA125, and CA15-3) were combined in a single multivariable logistic regression framework to assess their joint discriminatory ability. Three models were employed to estimate the diagnostic power of the combined scores for discriminating borderline/malignant tumors, and three models for discriminating only malignant tumors. For each model, both rounded and exact formula approaches were applied to calculate predicted probabilities. Separate models were developed for the borderline/malignant group and the malignant-only group, enabling direct comparison of biomarker performance across different diagnostic strata. The diagnostic performances of individual biomarkers and combined scores were evaluated using the area under the ROC curve (AUC), and the best cut-off value was calculated using the DeLong et al. method [44]. Sensitivity, specificity, and accuracy with a 95% confidence interval (CI) were calculated. A *p*-value of <0.05 was considered statistically significant.

## 3. Results

**Figure 1:**
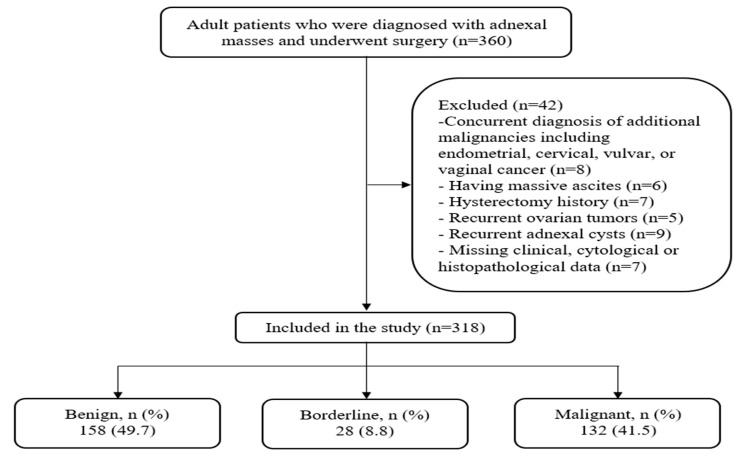
Flow diagram of the study.

During the study period, 318 patients were included in the study according to the inclusion and exclusion criteria. Of the patients, 158 had benign (49.7%), 28 had borderline (8.8%), and 132 had malignant (41.5%) tumors (Figure 1).

The median age was 51 years, and the majority of the cohort was postmenopausal (*n* = 209, 65.7%). Median age increased across groups (benign 49.0 years, borderline 51.5 years, malignant 57.0 years; *p* < 0.001). Postmenopausal status was more common in malignant cases (78.8% vs. 55.1% benign; *p* < 0.001). Comparing the levels of blood-derived traditional tumor markers, CA125 (*p* < 0.001), CA19-9 (*p* = 0.014), CA15-3 (*p* < 0.001), CEA (*p* = 0.003), LDH (*p* = 0.004), and procalcitonin (*p* = 0.031) as well as FAR (*p* < 0.001), distinguishes benign cases from malignant. Specifically, CA125 levels were significantly correlated with cytological findings, and CA125 successfully stratified borderline cases as benign and malignant (*p* < 0.05). FAR, CA15-3, and LDH were directly correlated with the categorization of borderline and malignant tumors (*p* < 0.05). Tumor size (median 9.00) was another factor aiding adnexal mass classification (Table 1). However, tumor size, procalcitonin, CEA, and CA19-9 were inefficient in distinguishing borderline tumors from benign masses (*p* > 0.05 for all).

Among the study group, adnexal masses were marginally represented as of epithelial origin (81.1%), and the group mainly consisted of masses of serous (46.5%), mucinous (20.1%), and endometrioid (9.7%) epithelial origin (Table 2). Majority of borderline tumors presented at FIGO stage I (96.4%), whereas malignant tumors had a broader stage distribution: stage I (28.0%), stage II (17.4%), stage III (32.6%), and stage IV (22.0%). Pelvic and para-aortic lymph node involvement and distant metastases occurred in 42.4% and 34.1% of malignant cases (Table 3).

Table 4 outlines the diagnostic performance of FAR and conventional serologic markers. The ROC analysis showed that FAR presented significant performance (0.735 vs. 0.767) at a cut-off > 11.51 (*p* < 0.001) in identifying borderline/malignant versus malignant lesions, respectively. Similarly, CA125 (0.808 vs. 0.813), CA19-9 (0.603 vs. 0.577), CA15-3 (0.785 vs. 0.820), CEA (0.606 vs. 0.600), and LDH (0.584 vs. 0.616) levels were strongly linked to malignant lesions (*p* < 0.05), whereas CA125 and CA15-3 presented the top two AUCs, followed by FAR.

**Figure 2:**
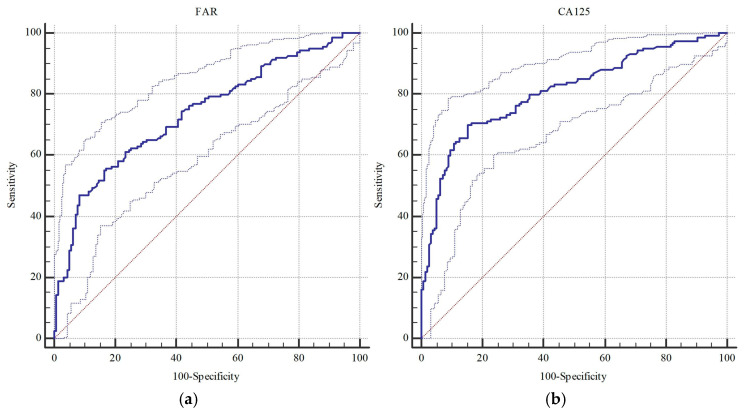

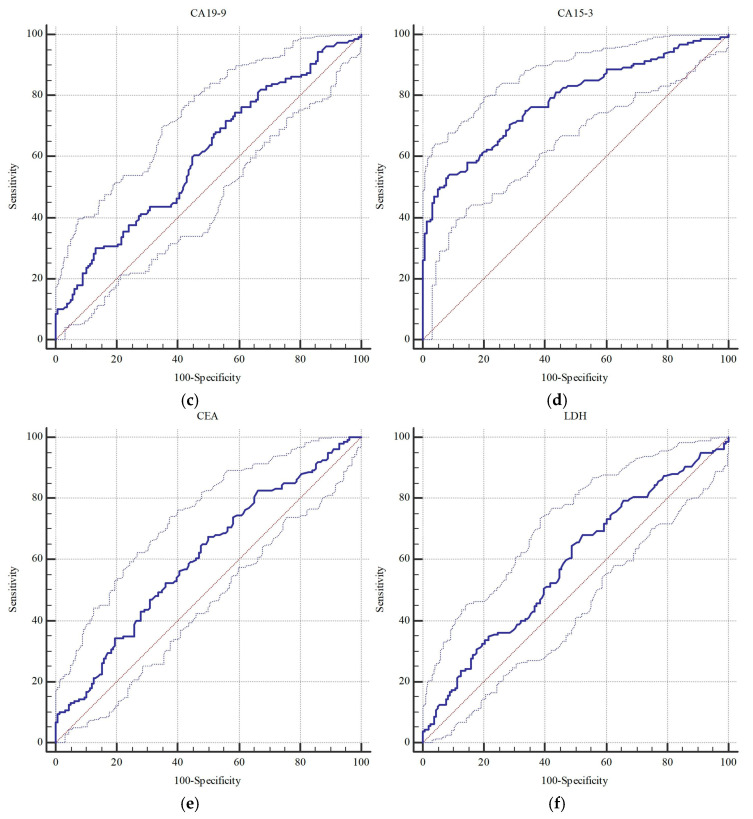
ROC curves of (**a**) FAR, (**b**) CA125, (**c**) CA19-9, (**d**) CA15-3, (**e**) CEA, and (**f**) LDH in estimating borderline or malignant tumors. Solid lines denote the ROC curves; dotted lines indicate the 95% confidence intervals.

**Figure 3:**
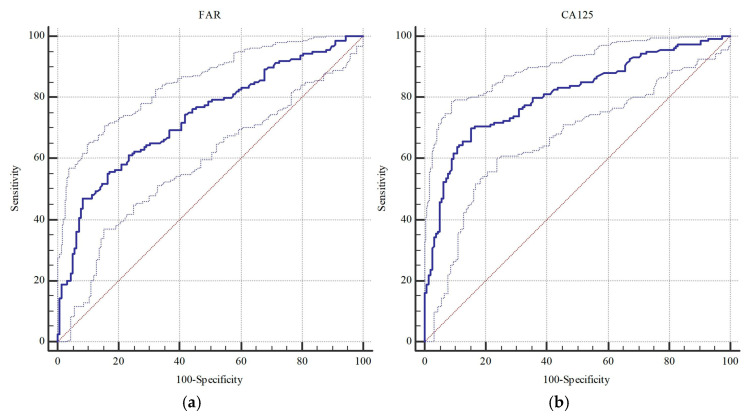

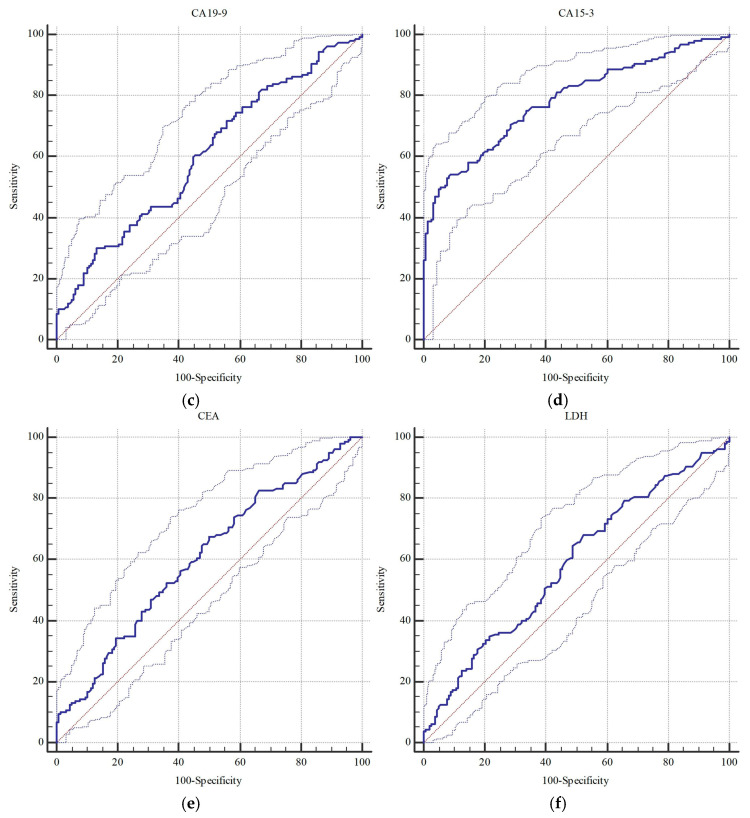
ROC curves of (**a**) FAR, (**b**) CA125, (**c**) CA19-9, (**d**) CA15-3, (**e**) CEA, and (**f**) LDH in estimating only malignant tumors. Solid lines denote the ROC curves; dotted lines indicate the 95% confidence intervals.

Among all parameters, CA125 (>49.9 U/mL) showed the best overall accuracy (77.4%) by achieving 70.0% sensitivity and 84.8% specificity. Cytology, while presenting perfect specificity (100%), was able to detect only less than half of the borderline/malignant cases (46.2% sensitivity). Similarly, sensitivity of FAR (>11.51) was lower than 50% (46.9%), but it showed near-perfect specificity (91.8%) and acceptable accuracy (69.2%). When focusing solely on malignancies (Table 5 and Table 6), CA125 again presented the best sensitivity (76.5%) and accuracy (79.3%), while cytology remained highly specific (99.5%) but only moderately sensitive (55.3%). FAR in this context showed 53.0% sensitivity, 90.3% specificity, and 74.8% accuracy, closely related to cytology. The ROC curves illustrate two groups of parameters consisting of CA125, CA15-3, and FAR (AUCs~0.81–0.82), and CA19-9, CEA, and LDH (AUC~0.735–0.767), achieving better vs. less performance in identification of borderline cases, respectively (Figure 2 and Figure 3).

In the multivariable logistic regression analysis including FAR, CA125, CA15-3, and menopausal status, all three biomarkers remained independent predictors of malignant pathology in both the borderline/malignant and malignant-only groups. Due to multicollinearity between age and menopausal status, age was excluded from the models (Table 7).

To improve diagnostic accuracy, we developed multivariable logistic regression models combining FAR, CA125, and CA15-3 to differentiate borderline/malignant tumors from benign adnexal masses (Supplementary Tables S1–S3). For each model, two formulas were derived using either rounded or exact regression coefficients. Among these, Formula 5·[(FAR × 1.2) + CA125 + CA15-3] achieved the highest AUC of 0.840 (95% CI 0.795–0.878) for borderline/malignant tumors, with no significant difference between rounded and exact coefficients (*p* = 0.864). This formula demonstrated a sensitivity of 71.3%, specificity of 84.2%, and overall accuracy of 77.7%, outperforming individual biomarkers and preoperative cytology.

We applied a similar modeling approach for malignant-only tumors (Supplementary Tables S4–S6). Formula 11·[(FAR × 1.2) + CA125 + (CA15-3 × 1.1)] achieved the highest performance, with an AUC of 0.851 (95% CI 0.806–0.889), sensitivity of 78.8%, specificity of 80.7%, and accuracy of 79.9%. The comparison of AUCs showed that combining biomarkers consistently improved discrimination compared with individual markers alone.

Overall, these findings suggest that integrating FAR with CA125 and CA15-3 enhances preoperative risk stratification for both borderline/malignant and malignant tumors. The rounded formulas provide a practical tool for clinical application, allowing for more accurate patient counseling and surgical planning while maintaining simplicity for routine use. This highlights that integrating FAR with CA125 and CA15-3 enhances preoperative risk stratification beyond single-marker assessment.

## 4. Discussion

Our findings demonstrate that the preoperative FAR was a valuable factor combined with conventional diagnostic tools in stratifying adnexal masses, particularly for identifying borderline tumors. Although CA125 and CA15-3 yielded marginally higher AUCs, FAR’s high specificity (>90%) makes it especially useful for ruling in non-benign pathology. These findings provide an alternative approach based on the combination of FAR with serologic biomarkers. Multivariable logistic regression models combining FAR, CA125, and CA15-3 demonstrated superior diagnostic accuracy for both borderline/malignant and malignant-only tumors compared with individual biomarkers. Notably, the three-marker models achieved the highest AUCs, and rounding regression coefficients did not compromise performance, supporting the clinical applicability of simplified risk stratification formulas. This is critical for surgical planning. Patients with borderline lesions often require fertility-sparing or conservative staging procedures. Therefore, distinguishing them from benign cysts preoperatively can optimize operative planning.

Adnexal masses are common in women. The incidence is largely affected by age and life stages, where major hormonal changes occur. In a larger Dutch cohort consisting of 11,595 patients, it was found that the incidence reaches 0.15% in adult women, and 7.7% of all cases were either malignant or borderline malignant [2]. A smaller cohort study (*n* = 187) found that 45.5% of the cases were in menopause, and 67.9% of those cases were malignant, whereas only 32.1% were malignant in premenopause women [45]. In another study, 60.7% of the patients with adnexal masses were postmenopausal, and the incidence of malignancy was significantly increased with age and postmenopausal status [46]. Comparable with the literature, our cohort mostly consisted of postmenopausal women (65.7%). The malignant lesion incidence was significantly associated with older age. The benign group had a mean age of 49 years old and the malignant group a mean age of 57 years old.

Serologic tumor markers, CA125, CA15-3, CA15-5, and CEA, are common cancer biomarkers. Studies primarily evaluating the efficacy of CA125, particularly in ovarian carcinoma, found moderate sensitivity (65.9–78.9%) and high specificity (76.6–86.9%) for distinguishing malignant tumors from benign adnexal masses [47,48]. However, using CA125 levels alone to stratify lesions was not recommended, since CA125 is detected in relatively increased levels in premenopausal women [11,49] and in a variety of conditions such as pregnancy, inflammation due to endometriosis, and abscesses [11,50].

In concordance with the literature, our results showed that CA125 was the best biomarker for stratifying borderline/malignant cases, with a sensitivity of 70.0% and 84.8% specificity, and for distinguishing malignant from benign lesions, with a sensitivity of 76.5% and 81.2%. Another marker, CA15-3, determined the malignancy of some adnexal masses with high specificity (96.1–98.2%) but low sensitivity (26.3–34.1%), particularly in postmenopause women [47,48]. Studies have shown that combining CA15-3 with CA125 improves accuracy, especially in levels above 44.5 U/mL [51], but its power remains limited [47,48]. Similarly, in our study, CA15-3 alone presented the second-best specificity and sensitivity. However, CA15-3 was poor at stratifying borderline/malignant cases from benign lesions, with a particularly low sensitivity. A recent study from Ruma et al. found that a rather poor serologic marker, CA19-9, can predict malignancy in ovarian masses when evaluated within the patients’ ascites or cyst fluid [52]. Other studies focusing on the serum levels of CA19-9 and CEA have shown that the biomarkers are solely able to ascertain metastatic tumors [53,54]. CEA additionally distinguishes mucinous-type ovarian tumors [54]. And, even combined with CA125, it presented poor capability to stratify borderline cases [53,54]. Well aligned with these studies, our findings suggested limited power of both CA19-9 and CEA serologic biomarkers for borderline cases.

FAR represents systemic inflammation and has been proposed as a diagnostic and prognostic marker across various pathologies [55]. Fibrinogen, an acute-phase reactant synthesized by the liver, is upregulated in response to pro-inflammatory cytokines such as IL-6 and IL-1β. A further immunological cascade boosts IL-6 and IL-1β levels, causing increased blood vessel permeability, leading to transcapillary albumin loss and hypoalbuminemia [56,57]. Various clinical studies, from cardiac diseases [27,28] and diabetes-associated kidney disease [32] to solid cancer [29,30,31], studied FAR primarily as a prognostic marker. However, its diagnostic role, particularly in distinguishing borderline adnexal lesions, remains underexplored. Our findings showed that FAR alone identifies only about half of borderline cases (sensitivity: 47%), but its more than 90% specificity complements CA125, which solely provides higher sensitivity (70%) but lower specificity (85%).

The Risk of Ovarian Malignancy Algorithm (ROMA) combines CA125 and HE4 levels with menopausal status to assess ovarian cancer risk. While ROMA has demonstrated high diagnostic accuracy, particularly in postmenopausal women [58], the performance of ROMA can vary based on patient demographics and tumor characteristics. HE4 alone has shown superior specificity compared to CA125, especially in distinguishing ovarian cancer from benign conditions [59]. In our study, FAR demonstrated comparable diagnostic performance to ROMA and HE4, suggesting that it could serve as an effective standalone biomarker or in combination with existing models to enhance diagnostic accuracy in evaluating adnexal masses.

By combining FAR with CA125 and potentially CA15-3, we foresee a diagnostic panel that could boost overall sensitivity to >85% while preserving specificity above 85%, offering balanced performance for preoperative stratification. This combinatory approach will provide considerable sensitivity and specificity. The combined FAR, CA125, and CA15-3 formulas can serve as practical decision-support tools in the preoperative evaluation of adnexal masses. High formula scores may identify patients at increased risk of borderline or malignant pathology, guiding surgical planning and fertility-sparing strategies [16]. When used alongside ultrasonography and clinical assessment, these biomarker combinations enable more accurate risk stratification, potentially reducing unnecessary surgeries and optimizing resource utilization [34].

A key strength of this study is the inclusion of a relatively large and well-characterized cohort with solid histopathological classification, allowing for comparison of serologic markers across benign, borderline, and malignant adnexal masses. Additionally, the real-world setting and use of routinely available laboratory parameters improved the clinical applicability of the findings. However, the retrospective single-center design may limit generalizability. Another important limitation is the relatively small size of the borderline tumor subgroup (*n* = 28), which may reduce the statistical power and limit the precision of diagnostic performance estimates for this group. Further multicenter prospective studies are warranted to validate the diagnostic utility of FAR and its integration into existing risk models. The FAR cut-off value (>11.51) was determined based on ROC curve analysis in our cohort, which may introduce a risk of overfitting and limit generalizability to external populations. Additionally, no internal validation (e.g., bootstrap or cross-validation) was performed for the multivariable logistic regression models, which should be considered when interpreting the reported AUCs and diagnostic performance.

## 5. Conclusions

FAR may provide complementary information to conventional biomarkers, as its inflammatory basis reflects aspects of the tumor microenvironment that are not captured by CA125 or CA15-3. While FAR alone shows limited sensitivity, its high specificity makes it more suitable as part of a combined biomarker strategy rather than as a standalone test. When integrated with CA125 and CA15-3, FAR improved the balance between sensitivity and specificity, suggesting a potential role in refining preoperative risk stratification of adnexal masses. Nevertheless, given the retrospective single-center design and the small number of borderline cases, these findings should be regarded as exploratory, and prospective multicenter validation is required before FAR can be incorporated into routine clinical practice.

## Figures and Tables

**Table 1:** Demographics and clinical features of the patients.

Variables	All Patients (*n* = 318)	Patients with Benign Masses (*n* = 158)	Patients with Borderline Tumors (*n* = 28)	Patients with Malignant Tumors (*n* = 132)	*p*
Age (year), Median (IQR)	51.0 (44.0–60.0)	49.0 (41.0–57.0)	51.5 (42.0–60.5)	57.0 (49.0–65.0)	<0.001 ^a^
Postmenopausal, *n* (%)	209 (65.7)	87 (55.1)	18 (64.3)	104 (78.8)	<0.001 ^b^
Fibrinogen (g/L), Median (IQR)	3.80 (3.24–4.60)	3.48 (3.07–4.08)	3.38 (3.09–4.48)	4.42 (3.61–5.66)	<0.001 ^a^
Albumin (g/L), Median (IQR)	43.0 (38.0–46.0)	45.0 (40.7–47.0)	44.0 (38.7–45.0)	40.0 (32.0–44.0)	<0.001 ^a^
FAR, Median (IQR)	8.97 (7.23–12.30)	7.85 (6.71–9.60)	8.56 (6.59–11.01)	11.72 (8.46–15.17)	<0.001 ^a^
CA125	32.80 (14.30–189.00)	17.45 (10.50–34.35)	28.50 (19.97–154.75)	185.50 (51.15–746.75)	<0.001 ^a^
CA19-9	13.90 (6.13–31.72)	10.60 (5.05–24.12)	19.60 (6.58–51.47)	14.80 (7.79–41.12)	0.006 ^a^
CA15-3	20.50 (14.85–33.10)	16.65 (12.00–22.42)	19.15 (15.72–23.22)	36.55 (21.55–99.95)	<0.001 ^a^
CEA	1.66 (1.01–2.66)	1.41 (0.90–2.32)	1.70 (1.24–2.55)	1.92 (1.13–3.00)	0.004 ^a^
AFP	2.20 (1.47–3.32)	2.13 (1.48–3.05)	2.76 (1.65–3.92)	2.22 (1.31–3.28)	0.167 ^a^
LDH	225.00 (181.75–147.05)	211.00 (175.75–262.50)	211.00 (180.50–232.75)	239.00 (193.50–320.25)	0.002 ^a^
Procalcitonin	0.29 (0.11–0.87)	0.22 (0.10–0.52)	0.49 (0.21–1.39)	0.37 (0.11–1.43)	0.005 ^a^
Tumor Size (cm), Median (IQR)	9.00 (5.00–15.00)	7.50 (4.50–12.00)	14.50 (8.00–20.75)	10.25 (5.00–16.00)	<0.001 ^a^
Bilaterality, *n* (%)	126 (39.6)	47 (29.7)	4 (14.3)	75 (56.8)	<0.001 ^b^

Note: IQR: interquartile range; FAR: fibrinogen-to-albumin ratio; CA 125: carbohydrate antigen 125; CA 19-9: carbohydrate antigen 19-9; CA 15-3: carbohydrate antigen 15-3; CEA: carcinoembryonic antigen; AFP: Alpha-fetoprotein; LDH: Lactate dehydrogenase. ^a^ Kruskal–Wallis Test was used. ^b^ Pearson’s chi-square test was used.

**Table 2:** Histological types of adnexal masses.

Histological Types (*n* = 318)		*n* (%)
Epithelial		258 (81.1)
	Serous	148 (46.5)
	Mucinous	64 (20.1)
	Endometrioid	31 (9.7)
	Clear cell	10 (3.1)
	Brenner	1 (0.3)
	Mixed epithelial	4 (1.3)
Mesenchymal		1 (0.3)
Sex-cord stromal		15 (4.7)
Germ cell		19 (6.0)
Tumor-like lesions		24 (7.5)
Mixed epithelial–germ cell		1 (0.3)

**Table 3:** Stage, lymph node invasion, and distant metastasis status of the borderline and malignant tumors.

Variables	Patients with Borderline Tumors (*n* = 28), *n* (%)	Patients with Malignant Tumors (*n* = 132), *n* (%)
Stage		
I	27 (96.4)	37 (28.0)
II	1 (3.6)	23 (17.4)
III	0 (0.0)	43 (32.6)
IV	0 (0.0)	29 (22.0)
PPLNI	-	56 (42.4)
Distant metastasis	-	45 (34.1)

Note: PPLNI: pelvic and para-aortic lymph node involvement.

**Table 4:** ROC analysis results of tumor markers in estimating borderline/malignant and malignant tumors.

Markers (*n* = 318)	Borderline/Malignant			Malignant		
	AUC (95% CI)	*p* ^a^	Cut-Off	AUC (95% CI)	*p* ^a^	Cut-Off
FAR	0.735 (0.683–0.783)	<0.001	>11.51	0.767 (0.717–0.812)	<0.001	>11.51
CA 125	0.808 (0.761–0.850)	<0.001	>49.9	0.813 (0.766–0.855)	<0.001	>49.9
CA 19-9	0.603 (0.546–0.657)	0.001	>34.8	0.577 (0.521–0.632)	0.018	>8.4
CA 15-3	0.785 (0.736–0.829)	<0.001	>29.8	0.820 (0.773–0.861)	<0.001	>29.5
CEA	0.606 (0.550–0.660)	<0.001	>1.4	0.600 (0.544–0.654)	0.002	>2.1
LDH	0.584 (0.528–0.639)	0.008	>211.0	0.616 (0.560–0.669)	<0.001	>211.0

Note: AUC: area under ROC curve; CI: confidence interval; FAR: fibrinogen-to-albumin ratio; CA 125: carbohydrate antigen 125; CA 19-9: carbohydrate antigen; CA 15-3: carbohydrate antigen 15-3; CEA: carcinoembryonic antigen; LDH: Lactate dehydrogenase. ^a^ Delong et al. method was used.

**Table 5:** Cross-tabulation of preoperative cytology and tumor markers for the assessment of borderline/malignant tumors.

Parameters (*n* = 318)		Histopathological Findings		Sensitivity (95% CI)	Specificity (95% CI)	Accuracy (95% CI)
		Benign	Borderline/Malignant			
Cytology	Benign	158 (49.7)	86 (27.0)	46.2 (38.3–54.3)	100.0 (97.7–100.0)	73.0 (67.7–77.8)
	Malignant	0 (0.0)	74 (23.3)			
FAR	≤11.51	145 (45.6)	85 (26.7)	46.9 (39.0–54.9)	91.8 (86.3–95.5)	69.2 (63.8–74.2)
	>11.51	13 (4.1)	75 (23.6)			
CA 125	≤49.9	134 (42.1)	48 (15.1)	70.0 (62.3–77.0)	84.8 (78.2–90.0)	77.4 (72.4–81.8)
	>49.9	24 (7.5)	112 (35.2)			
CA 19-9	≤34.8	137 (43.1)	112 (35.2)	30.0 (23.0–37.7)	86.7 (80.4–91.6)	58.2 (52.5–63.7)
	>34.8	21 (6.6)	48 (15.1)			
CA 15-3	≤29.8	146 (45.9)	75 (23.6)	53.1 (45.1–61.0)	92.4 (87.1–96.0)	72.6 (67.4–77.5)
	>29.8	12 (3.8)	85 (26.7)			
CEA	≤1.4	79 (24.8)	52 (16.4)	67.5 (59.7–74.7)	50.0 (42.0–58.0)	58.8 (53.2–64.3)
	>1.4	79 (24.8)	108 (34.0)			
LDH	≤211.0	81 (25.5)	57 (17.9)	64.4 (56.4–71.8)	51.3 (43.2–59.3)	57.9 (52.2–63.3)
	>211.0	77 (24.2)	103 (32.4)			

Note: CI: confidence interval; FAR: fibrinogen-to-albumin ratio; CA 125: carbohydrate antigen 125; CA 19-9: carbohydrate antigen; CA 15-3: carbohydrate antigen 15-3; CEA: carcinoembryonic antigen; LDH: Lactate dehydrogenase.

**Table 6:** Cross-tabulation of preoperative cytology and tumor markers for the assessment of malignant tumors.

Parameters (*n* = 318)		Histopathological Findings		Sensitivity (95% CI)	Specificity (95% CI)	Accuracy (95% CI)
		Benign /Borderline	Malignant			
Cytology	Benign	185 (58.2)	59 (18.6)	55.3 (46.4–64.0)	99.5 (97.0–100.0)	81.1 (76.4–85.3)
	Malignant	1 (0.3)	73 (23.0)			
FAR	≤11.51	168 (52.8)	62 (19.5)	53.0 (44.2–61.8)	90.3 (85.1–94.2)	74.8 (69.7–79.5)
	>11.51	18 (5.7)	70 (22.0)			
CA 125	≤49.9	151 (47.5)	31 (9.7)	76.5 (68.4–83.5)	81.2 (74.8–86.5)	79.3 (74.4–83.6)
	>49.9	35 (11.0)	101 (31.8)			
CA 19-9	≤8.4	80 (25.2)	36 (11.3)	72.7 (64.3–80.1)	43.0 (35.8–50.5)	55.4 (49.7–60.9)
	>8.4	106 (33.3)	96 (30.2)			
CA 15-3	≤29.5	170 (53.5)	49 (15.4)	62.9 (54.0–71.1)	91.4 (86.4–95.0)	79.6 (74.7–83.9)
	>29.5	16 (5.0)	83 (26.1)			
CEA	≤2.1	133 (41.8)	76 (23.9)	42.4 (33.9–51.3)	71.5 (64.4–77.9)	59.4 (53.8–64.9)
	>2.1	53 (16.7)	56 (17.6)			
LDH	≤211.0	96 (30.2)	42 (13.2)	68.2 (59.5–76.0)	51.6 (44.2–59.0)	58.5 (52.9–94.0)
	>211.0	90 (28.3)	90 (28.3)			

Note: CI: confidence interval; FAR: fibrinogen-to-albumin ratio; CA 125: carbohydrate antigen 125; CA 19-9: carbohydrate antigen 19-9; CA 15-3: carbohydrate antigen 15-3; CEA: carcinoembryonic antigen; LDH: Lactate dehydrogenase.

**Table 7:** Multivariable logistic regression analysis of FAR, CA125, and Ca15-3 in estimating malignant tumors.

Variables ^a^		Borderline/Malignant		Malignant	
		OR (95% CI)	*p*	OR (95% CI)	*p*
FAR		1.140 (1.053–1.234)	0.001	1.165 (1.077–1.260)	<0.001
CA125		1.003 (1.001–1.005)	0.003	1.003 (1.001–1.005)	0.005
CA15-3		1.048 (1.021–1.075)	<0.001	1.060 (1.021–1.089)	<0.001
Menopausal status	Pre/Perimenopausal	R		R	
	Postmenopausal	2.262 (1.225–4.174)	0.009	2.815 (1.375–5.761)	0.005

Note: OR: Odds Ratio: confidence interval; FAR: fibrinogen-to-albumin ratio; CA 125: carbohydrate antigen 125; CA 15-3: carbohydrate antigen 15-3. ^a^ Two multivariable logistic regression models with either method were employed using FAR, CA125, CA15-3, age, and menopausal status. Since there was a multicollinearity between age and menopausal status, age was excluded from both models.

## Data Availability

The original contributions presented in this study are included in the article. Further inquiries can be directed to the corresponding author.

## Supplementary Materials

The following supporting information can be downloaded at https://www.mdpi.com/article/10.3390/diagnostics15182372/s1, Table S1. Multivariable logistic regression analysis of FAR, CA125 and Ca15-3 in estimating borderline/malignant masses; Table S2. ROC analysis results of model formulas derived from multivariable logistic regression analysis to combine FAR, CA125 and or CA15-3 in estimating borderline/malignant masses; Table S3. Cross-tabulation of p values of statistical comparisons of AUCs of FAR, CA125, CA15-3, and rounded formulas derived from multivariable logistic regression analysis in estimating borderline/malignant masses; Table S4. Multivariable logistic regression analysis of FAR, CA125 and Ca15-3 in estimating malignant masses.; Table S5. Model formulas derived from multivariable logistic regression analysis to combine FAR, CA125 and or CA15-3 in estimating malignant masses; Table S6. Cross-tabulation of p-values of statistical comparisons of AUCs of FAR, CA125, CA15-3, and rounded formulas derived from multivariable logistic regression analysis in estimating malignant masses; Table S7. Cross-tabulation of combined scores for the assessment of borderline/malignant tumors; Table S8. Cross-tabulation of combined scores for the assessment of malignant tumors; Figure S1. ROC curves of FAR, CA125, CA15-3, and rounded formulas derived from multivariable logistic regression analysis in estimating borderline/malignant masses; Figure S2. ROC curves of FAR, CA125, CA15-3, and rounded formulas derived from multivariable logistic regression analysis in estimating malignant masses.
