# Supplementary material for: Diagnostic Power of the Fibrinogen-to-Albumin Ratio for Estimating Malignancy in Patients with Adnexal Masses: A Methodological Study
Source: Diagnostics (Basel). 2025 Sep 18;15(18):2372. doi: 10.3390/diagnostics15182372 (PMC12469132; doi:10.3390/diagnostics15182372)
Supplement: Supplementary file 1 [file diagnostics-15-02372-s001.zip › diagnostics-3808391-supplementary.pdf]

Supplementary Table S1. Multivariable logistic regression analysis of FAR, CA125 and Ca15-3 in estimating borderline/malignant masses.

Model No	Variables in the model	B	S.E.	Wald	df	p	Exp(B)	95% CI for Exp (B)
Model 1	FAR	0.186	0.040	21.785	1	**<0.001**	1.204	1.114-1.302
	CA125	0.005	0.001	18.470	1	**<0.001**	1.005	1.002-1.007
	Constant	-2.438	0.402	36.758	1	**<0.001**	0.087	
Model 2	FAR	0.160	0.040	15.822	1	**<0.001**	1.173	1.084-1.269
	CA15-3	0.057	0.012	21.514	1	**<0.001**	1.059	1.033-1.084
	Constant	-3.099	0.447	48.051	1	**<0.001**	0.045	
Model 3	FAR	0.152	0.041	13.482	1	**<0.001**	1.164	1,073-1.262
	CA125	0.003	0.001	8.270	1	**0.004**	1.003	1,001-1.005
	CA15-3	0.046	0.013	13.516	1	**<0.001**	1.048	1,022-1.074
	Constant	-3.064	0.459	44.603	1	**<0.001**	0.047	

Note: B: Regression coefficient, S.E.: Standard error, Wald: Wald chi-square test statistic, df: Degrees of freedom, Exp(B): Exponentiated coefficient (Odds Ratio, OR), CI: Confidence Interval, FAR: Fibrinogen-albumin ratio, CA 125: Carbohydrate antigen 125, CA 15-3: Carbohydrate antigen 15-3.

Supplementary Table S2. ROC analysis results of model formulas derived from multivariable logistic regression analysis to combine FAR, CA125 and or CA15-3 in estimating borderline/malignant masses.

Model No	Formula No[a]	Formula	AUC (95% CI)	p^b
Model 1	Formula 1	(FAR x 1.2) + CA125 + 0.1	0.822 (0.776-0.863)	0.999
	Formula 2	(FAR x 1.204) + (CA125 x 1.005) + 0.087	0.822 (0.776-0.863)	
Model 2	Formula 3	(FAR x 1.2) + (CA 15-3 x 1.1)	0.807 (0.759-0.849)	0.856
	Formula 4	(FAR x 1.173) + (CA 15-3 x 1.059) + 0.045	0.807 (0.759-0.849)	
Model 3	Formula 5	(FAR x 1.2) + CA125 + CA 15-3	0.840 (0.795-0.878)	0.864
	Formula 6	(FAR x 1.164) + (CA125 x 1.003) + (CA 15-3 x 1.048) + 0.047	0.840 (0.795-0.878)	

[a]The coefficients in formulas 1, 3 and 5 have been rounded to the nearest number.

[b]Delong et al. method was used to compare diagnostic performance of combined formulas in each model.
Note: CI: Confidence Interval, FAR: Fibrinogen-albumin ratio, CA 125: Carbohydrate antigen 125, CA 15-3: Carbohydrate antigen 15-3.

Supplementary Table S3. Cross-tabulation of p values of statistical comparisons of AUCs of FAR, CA125, CA15-3, and rounded formulas derived from multivariable logistic regression analysis in estimating borderline/malignant masses[a]

	FAR	CA125	CA15-3	Formula 1	Formula 3	Formula 5
**FAR**	-					
**CA125**	**0.025**	-				
**CA15-3**	0.123	0.406	-			
**Formula 1**	**0.003**	**0.019**	0.168	-		
**Formula 3**	**0.005**	0.952	**0.021**	0.531	-	
**Formula 5**	**<0.001**	**0.002**	**0.016**	**0.016**	0.110	-

[a]Delong et al. method was used to compare diagnostic performance of biomarkers and formulas.

Note: FAR: Fibrinogen-albumin ratio, CA 125: Carbohydrate antigen 125, CA 15-3: Carbohydrate antigen 15-3.

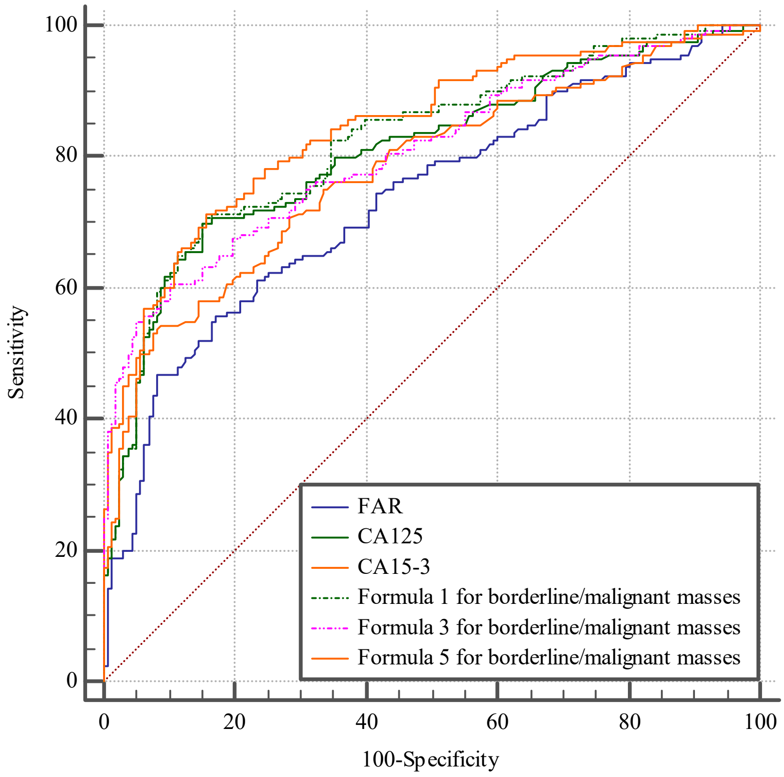

Supplementary Figure S1. ROC curves of FAR, CA125, CA15-3, and rounded formulas derived from multivariable logistic regression analysis in estimating borderline/malignant masses

Supplementary Table S4. Multivariable logistic regression analysis of FAR, CA125 and Ca15-3 in estimating malignant masses.

Model No	Variables in the model	B	S.E.	Wald	df	p	Exp(B)	95% CI for Exp (B)
Model 4	FAR	0.211	0.039	28.568	1	**<0.001**	1.235	1.143-1.334
	CA125	0.004	0.001	22.006	1	**<0.001**	1.004	1.002-1.006
	Constant	-3.165	0.424	55.660	1	**<0.001**	0.042	
Model 5	FAR	0.177	0.040	19.542	1	**<0.001**	1.194	1.104-1.292
	CA15-3	0.066	0.001	18.470	1	**<0.001**	1.069	1.042-1.096
	Constant	-4.049	0.491	67.994	1	**<0.001**	0.017	
Model 6	FAR	0.171	0.041	17.074	1	**<0.001**	1.187	1.094-1.287
	CA125	0.002	0.001	7.227	1	**0.007**	1.002	1.001-1.004
	CA15-3	0.057	0.013	18.399	1	**<0.001**	1.058	1.031-1.086
	Constant	-4.026	0.504	63.727	1	**<0.001**	0.018	

Note: B: Regression coefficient, S.E.: Standard error, Wald: Wald chi-square test statistic, df: Degrees of freedom, Exp(B): Exponentiated coefficient (Odds Ratio, OR), CI: Confidence Interval, FAR: Fibrinogen-albumin ratio, CA 125: Carbohydrate antigen 125, CA 15-3: Carbohydrate antigen 15-3.

Supplementary Table S5. Model formulas derived from multivariable logistic regression analysis to combine FAR, CA125 and or CA15-3 in estimating malignant masses.

Model No	Formula No [a]	Formula	AUC (95% CI)	*p* [b]
Model 4	Formula 7	(FAR x 1.2) + CA125	0.830 (0.785-0.870)	0.499
	Formula 8	(FAR x 1.235) + (CA125 x 1.004) + 0.042	0.831 (0.785-0.870)	
Model 5	Formula 9	(FAR x 1.2) + (CA 15-3 x 1.1)	0.848 (0.803-0.885)	0.892
	Formula 10	(FAR x 1.194) + (CA 15-3 x 1.069) + 0.017	0.848 (0.804-0.886)	
Model 6	Formula 11	(FAR x 1.2) + CA125 + (CA 15-3 x 1.1)	0.851 (0.807-0.889)	0.874
	Formula 12	(FAR x 1.187) + (CA125 x 1.002) + (CA 15-3 x 1.058) + 0.018	0.850 (0.806-0.888)	

[a]The coefficients in formulas 1, 3 and 5 have been rounded to the nearest number.

[b]Delong et al. method was used to compare diagnostic performance of combined formulas in each model.

Note: CI: Confidence Interval, FAR: Fibrinogen-albumin ratio, CA 125: Carbohydrate antigen 125, CA 15-3: Carbohydrate antigen 15-3.

Supplementary Table S6. Cross-tabulation of p values of statistical comparisons of AUCs of FAR, CA125, CA15-3, and rounded formulas derived from multivariable logistic regression analysis in estimating malignant masses[a]

	FAR	CA125	CA15-3	Formula 7	Formula 9	Formula 11
**FAR**	-					
**CA125**	0.166	-				
**CA15-3**	0.097	0.811	-			
**Formula 7**	**0.035**	0.203	0.702	-		
**Formula 9**	**0.003**	**0.008**	0.164	0.478	-	
**Formula 11**	**0.002**	**<0.001**	**0.004**	**0.009**	0.855	-

[a]Delong et al. method was used to compare diagnostic performance of biomarkers and formulas.

Note: FAR: Fibrinogen-albumin ratio, CA 125: Carbohydrate antigen 125, CA 15-3: Carbohydrate antigen 15-3.

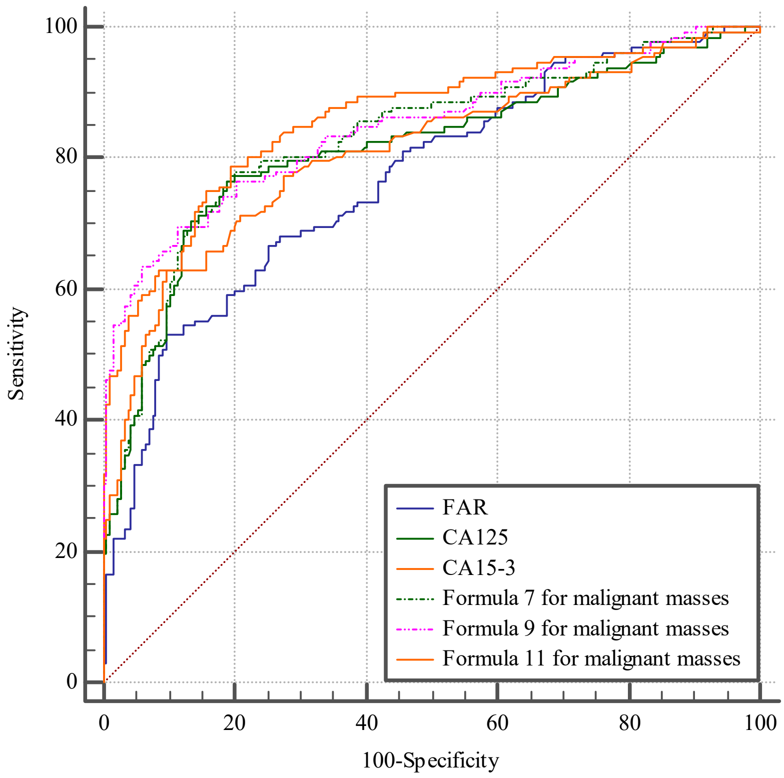

Supplementary Figure S2. ROC curves of FAR, CA125, CA15-3, and rounded formulas derived from multivariable logistic regression analysis in estimating malignant masses

Supplementary Table S7. Cross-tabulation of combined scores for the assessment of borderline/malignant tumors.

Formula 5	Cut-off	Sensitivity (95% CI)	Specificity (95% CI)	Accuracy (95% CI)
(FAR x 1.2) + CA125 + CA15-3	>83.92	71.3 (63.6-78.1)	84.2 (77.5-89.5)	77.7 (72.7-82.1)

Note: CI: Confidence interval, FAR: Fibrinogen-to-Albumin Ratio, CA125: Carbohydrate antigen 125, CA15-3: Carbohydrate antigen 15-3.

Supplementary Table S8. Cross-tabulation of combined scores for the assessment of malignant tumors.

Formula 11	Cut-off	Sensitivity (95% CI)	Specificity (95% CI)	Accuracy (95% CI)
(FAR x 1.2) + CA125 + (CA15-3 x 1.1)	>86.41	78.8 (70.8-85.4)	80.7 (74.2-86.1)	79.9 (75.0-84.1)

Note: CI: Confidence interval, FAR: Fibrinogen-to-Albumin Ratio, CA125: Carbohydrate antigen 125, CA15-3: Carbohydrate antigen 15-3.
